# Effect of linoleic acid on ischemic heart disease and its risk factors: a Mendelian randomization study

**DOI:** 10.1186/s12916-019-1293-x

**Published:** 2019-03-14

**Authors:** Jie V. Zhao, C. Mary Schooling

**Affiliations:** 1 School of Public Health, Li Ka Shing Faculty of Medicine, The University of Hong Kong, 1/F, Patrick Manson Building, 7 Sassoon Road, Hong Kong, SAR China; 2 School of Public Health and Health Policy, City University of New York, New York, NY USA

**Keywords:** Unsaturated fatty acid, Mendelian randomization, Cardiovascular disease, Diabetes

## Abstract

**Background:**

The role of n-6 polyunsaturated fatty acids (PUFAs) in ischemic heart disease (IHD) is controversial, and dietary guidelines vary. Observationally, lower saturated fat intake and higher intake of vegetable oils rich in linoleic acid (LA), the main n-6 PUFA, is associated with lower IHD and diabetes; however, randomized controlled trials have not fully corroborated these benefits. We assessed how genetically predicted LA affected IHD and its risk factors, including diabetes, lipids, and blood pressure. We also assessed the role of LA in reticulocyte count, the red blood cell precursor, which has recently been identified as a possible causal factor in IHD.

**Methods:**

Two-sample instrumental variable analysis with genetic instruments, i.e., Mendelian randomization, was used to obtain unconfounded estimates using genetic variants strongly (*p* value < 5 × 10^−8^) and solely associated with LA, applied to an IHD case (*n* ≤ 76,014)-control (*n* ≤ 264,785) study (mainly based on the meta-analysis of CARDIoGRAMplusC4D 1000 Genomes and UK Biobank CAD SOFT GWAS), the DIAbetes Genetics Replication And Meta-analysis diabetes case (*n* = 26,676)-control (*n* = 132,532) study, lipids from the Global Lipids Genetics Consortium Results (*n* = 196,475), and reticulocyte count and blood pressure from the UK Biobank (*n* ≤ 361,194). A weighted median and Mendelian randomization Egger were used for sensitivity analysis.

**Results:**

Genetically predicted LA was not associated with IHD or systolic blood pressure. Genetically predicted higher serum LA was associated with lower diabetes (odds ratio (OR) 0.97 per percentage in total fatty acid increase in LA, 95% confidence interval (CI) 0.96 to 0.99) and lower lipids (low-density lipoprotein, high-density lipoprotein, and total cholesterol), but may be associated with higher diastolic blood pressure. The findings were robust to different single nucleotide polymorphism (SNP) selections, analytic methods, and correction for multiple testing.

**Conclusions:**

Our novel study suggests a benefit of LA for diabetes and lipids but no benefit for IHD, blood pressure, or reticulocyte count. Explicating these paradoxical findings would facilitate identification of effective new interventions for diabetes and IHD.

**Electronic supplementary material:**

The online version of this article (10.1186/s12916-019-1293-x) contains supplementary material, which is available to authorized users.

## Introduction

Cardiovascular disease (CVD) is the leading cause of mortality globally [1]. Lowering intake of saturated fat and replacing it with unsaturated fat has been a mainstay of heart health dietary advice for the last 30 years [2]. Randomized controlled trials (RCTs) have largely substantiated the cardiovascular benefits of monounsaturated fats, such as olive oil [3]. In contrast, controversy has arisen about the effects of n-6 polyunsaturated fatty acids (PUFAs), particularly linoleic acid (LA) which is a major constituent of widely used polyunsaturated vegetable oils, such as sunflower, corn, soybean, and cottonseed oils [4]. Over the last half century in the USA, with the shift of dietary fat sources toward polyunsaturated seed oils, mainly soybean oil [5], dietary LA intake has increased dramatically [5], and adipose tissue LA has more than doubled [6].

Observationally, dietary LA intake or serum LA is usually inversely associated with ischemic heart disease (IHD) [7] and its risk factors, including diabetes [8], low-density lipoprotein (LDL) cholesterol, total cholesterol, and blood pressure [9]. However, observational studies are difficult to interpret because of potential confounding by factors, such as socioeconomic position, lifestyle, and health status, as well as the difficulty of distinguishing between co-occurring dietary elements. The cholesterol-lowering effect of LA is well-established [10]; however, the effect of LA on IHD is inconsistent in meta-analyses of RCTs, showing a beneficial effect [4], or neutral effects [11, 12], as new analyses of RCTs from many years ago, such as the Sydney Diet Heart Study and the Minnesota Coronary Experiment [10, 11], have come to light. Recommendations for LA intake in dietary guidelines vary, from less than 4% to up to 10% of energy intake [8].

Comparing events according to different levels of genetically predicted LA, i.e., Mendelian randomization (MR), can provide unconfounded estimates in an observational setting, because the genetic variants are determined at conception and thus are not affected by key confounders in conventional observational studies, such as health status, socioeconomic position, and lifestyle [13]. MR has been applied to examine the effect of LA on colorectal cancer [14], but to our knowledge, no MR study has assessed the effect of LA on IHD. Using genetically instrumented LA from a genome-wide association study (GWAS) of n-6 PUFA [16], and a very large case-control study of IHD with extensive genotyping, we conducted an MR study to examine the independent role of LA in IHD. We similarly assessed the associations with its risk factors, including diabetes, lipids, and blood pressure, to identify if any associations with IHD were independent of these risk factors. We also examined its role in reticulocyte count, the red blood cell precursor, which has been recently identified as a possible causal factor for IHD [15].

## Methods

### Genetic instruments for LA

Genetic predictors, i.e., single nucleotide polymorphisms (SNPs) strongly (*p* value < 5 × 10^−8^) associated with LA, were obtained from GWAS of n-6 PUFA [16]. One hundred seventy-three genome-wide significant SNPs have been identified [16]. First, we used all SNPs reaching genome-wide significance and in genes relevant to the biological metabolism of n-6 PUFA, i.e., *FADS1*, *FADS2*, and *NTAN1* (Additional file 1: Table S1). Specifically, *FADS1* and *FADS2* encode fatty acid desaturases and the *NTAN1* gene regulates desaturase activity [16, 17]*.* The correlation between SNPs were obtained using LDlink [18], which can easily and efficiently interrogate correlation matrix in specific population groups. Second, we used the most significant three uncorrelated SNPs in GWAS, as previously described [14, 19]. We also used all the genome-wide significant SNPs and took account of their correlations using principal components analysis (PCA) [20].

The strength of each SNP was evaluated using the *F*-statistic, calculated using a well-established approximation [21]. A cutoff of 10 was used as a “rule of thumb” to distinguish between strong and weak instruments [22]. To ensure the SNPs predicting LA were not confounded with the outcome, we assessed their Bonferroni-corrected associations with key confounders, i.e., socioeconomic position (job type and Townsend index), and lifestyle factors (alcohol intake frequency, smoking status, and frequency of moderate and vigorous physical activity) in the UK Biobank. To ensure the selected SNPs were solely linked with the outcomes via LA, we also checked for pleiotropy. Specifically, we checked whether these SNPs are directly associated with IHD and its risk factors using three comprehensive curated genetic cross-reference systems, Ensembl [23], GWAS catalog [24], and PhenoScanner [25], which provide all well-established associations of known SNPs with their phenotypes, including sub-genome-wide associations, and excluded SNPs with these direct associations (potential pleiotropy). Where a SNP predicting LA was not available for the outcomes, we sought a highly correlated proxy (*r*^2^ ≥ 0.8).

### Genetic associations with IHD, diabetes, lipids, and blood pressure

Genetic associations with IHD were obtained from the largest publicly available IHD case (*n* ≤ 76,014)-control (*n* ≤ 264,785) study based on CARDIoGRAMplusC4D 1000 Genomes (cases = 60,801, control = 123,504), the Myocardial Infarction Genetics and CARDIoGRAM Exome, the UK Biobank SOFT CAD GWAS (cases = 10,801, controls = 137,371), and two small case (*n* = 4412)-control (*n* = 3910) studies giving up to 340,799 individuals in total. In CARDIoGRAMplusC4D 1000 Genomes, most of the participants (77%) are of European descent. IHD status was determined from clinical diagnosis, medical records and self-report of medication usage, procedures such as revascularization, and other evidence of stenosis such as from coronary angiography. In the UK Biobank, 502,713 individuals aged 40–69 years, mean age 56.5 years, were recruited from England, Scotland, and Wales between 2006 and 2010, with a median follow-up for 7.1 years. Ninety-four percent of the participants are of self-reported European ancestry, and 45.6% are men. The phenotype of cases included fatal or nonfatal myocardial infarction, chronic IHD, percutaneous transluminal coronary angioplasty or coronary artery bypass grafting, and angina. Controls were those who were free from case status.

Genetic associations with diabetes, adjusted for age, sex, and principal components, were obtained from the DIAbetes Genetics Replication And Meta-analysis (DIAGRAM), diabetes case (*n* = 26,676)-control (*n* = 132,532) study, mean age 54.7 years old, 44% men. Genetic associations with lipids (as inverse normal transformed effect sizes), including high-density lipoprotein (HDL) cholesterol, LDL cholesterol, and total cholesterol, adjusted for age, age^2^, and sex, were obtained from the Global Lipids Genetics Consortium Results including 188,577 participants of European descent and 7898 participants of non-European descent, mean age 55.2 years old. Genetic associations with blood pressure and reticulocyte count were provided by Neale Lab [26], in 361,194 White British (194,174 men [46%]). The study adjusted for age, age^2^, 20 principal components, sex, and interactions of sex with age and age^2^.

### Statistical analysis

We obtained associations of LA with IHD, diabetes, lipids, blood pressure, and reticulocyte count from two-sample instrumental variable analysis. We aligned the SNPs based on allele letter and allele frequency. We obtained the Wald estimate (ratio of the genetic association with IHD and its risk factors to the genetic association with LA) for each SNP. After excluding potentially pleiotropic SNPs, we used PCA for all genome-wide significant SNPs and all functionally relevant SNPs. PCA uses all the SNPs and does not suffer from numerical instabilities arising from the potentially arbitrary SNP selection or the genetic correlation matrix [20]. For the most significant three SNPs, we combined SNP-specific estimates using inverse variance weighting (IVW) with fixed or multiplicative random effects, which gives consistent point estimates [27, 28]. The latter was used only when heterogeneity existed, to account for the additional uncertainly [27]. The IVW estimate in summary data approximates the genetic score estimate in individual-level data [29].

To control for pleiotropy, we conducted several sensitivity analyses. First, we used a weighted median and MR Egger, which are more robust to pleiotropy. A weighted median method gives consistent estimates even when up to 50% of the information comes from invalid SNPs [30]. MR Egger is based on the assumption that the pleiotropic associations are independent from the genetic associations with the exposure [31]. Second, we checked whether the intercept from MR Egger was non-zero because this indicates that some of the genetic predictors might be acting other than via LA (i.e., directionally pleiotropic). Third, we used different SNP selection methods, i.e., functionally relevant SNPs, SNPs with top significance in GWAS, and all genome-wide significant SNPs. Fourth, we excluded potentially pleiotropic SNPs directly related to cardiovascular disease risk factors. Fifth, we tested the association of docosapentaenoic acid (DPA) with IHD to check for pleiotropy due to n-3 PUFA. The role of DPA in IHD has not been examined in RCTs, so we tested it in this MR study even though serum DPA may only correlate weakly with dietary intake [32]. The role of eicosapentaenoic acid (EPA) and docosahexaenoic acid (DHA) in major cardiovascular events has been assessed in RCTs [33–35], with generally null findings [34, 35], although potential benefits for myocardial infarction [34] and from a specific n-3 PUFA, icosapent ethyl, have been found [33]. These discrepancies may be due to differences in study design [36, 37] or subtle differences in the magnitude of their effects on apolipoprotein B [33, 38] which needs clarification in future studies comparing various n-3 PUFAs along with examination of specific mechanistic pathways. All statistical analyses were conducted using R version 3.4.4 (R Foundation for Statistical Computing, Vienna, Austria) and the R package “MendelianRandomization.” This analysis of publicly available data does not require ethical approval.

## Results

### Genetic instruments for LA

We obtained 173 genome-wide significant SNPs from GWAS of n-6 PUFA in 8631 adults of European ancestry, mean aged 60 years old, 55% women [16]; 167 were bi-allelic and available for the outcomes. Of the 167 SNPs, 47 SNPs were in genes functionally relevant to LA (*FADS1*, *FADS2*, and *NTAN1*) and used. Genetically predicted DPA was not associated with IHD (Table 1) and so should not generate pleiotropy.

**Table 1:** Mendelian randomization estimates of associations of genetically predicted linoleic acid with ischemic heart disease (IHD) and diabetes using different SNP selections

Exposure	Selection of SNPs	Method	#SNPs	IHD			Diabetes		
				OR	95% CI	*p*	OR	95% CI	*p*
Linoleic acid (% in total fatty acids)	Genome-wide significant and functionally relevant SNPs	PCA	47	1.01	0.95 to 1.08	0.71	*0.97*	*0.96 to 0.99*	*< 0.001*
	Most significant SNPs	IVW*	3	0.99	0.96 to 1.01	0.18	*0.97*	*0.95 to 0.99*	*0.001*
	All genome-wide significant SNPs	PCA	167	1.00	0.96 to 1.05	0.92	*0.98*	*0.96 to 0.99*	*0.001*
DPA (% in total fatty acids)	Most significant SNPs in GWAS of n-3 PUFA (rs780094 and rs3734398)	IVW^†^	2	1.10	0.77 to 1.59	0.60	0.45	0.10 to 2.02	0.30

*CI* confidence interval, *DPA* docosapentaenoic acid, *IVW* inverse variance weighting, *OR* odds ratio, *PCA* principal components analysis, *PUFA* polyunsaturated fatty acid

*IVW with fixed effects was used for diabetes, and IVW with random effects (heterogeneity test *p* value < 0.05) was used for IHD

^†^IVW with fixed effects was used for IHD, and IVW with random effects (heterogeneity test *p* value < 0.05) was used for diabetes

For comparison, we used three SNPs (rs174547 (*FADS1*), rs10740118 (*JMJD1C*), and rs16966952 (*NTAN1*)), with top significance in GWAS, as previously described [14, 19]. We also used all 167 bi-allelic genome-wide significant SNPs for IHD, diabetes, LDL cholesterol, and total cholesterol and used 141 SNPs for HDL cholesterol, diastolic blood pressure, and reticulocyte count after excluding 26 SNPs with direct associations with these risk factors. These SNPs were not excluded for IHD because they might be on the pathway.

All SNPs had an *F*-statistic > 10 and reached genome-wide significance. None of the selected SNPs was associated with key confounders in the UK Biobank (Additional file 1: Table S1).

### Associations with IHD, diabetes, lipids, blood pressure, and reticulocyte count

Genetically instrumented LA was not associated with IHD (Table 1). Genetically instrumented LA was associated with lower risk of diabetes (Table 1) and lower LDL cholesterol, HDL cholesterol, and total cholesterol (Table 2), robust to different SNP selections, analysis methods, and multiple testing correction (Tables 1, 2, and 3). There was no indication of directional pleiotropy except for systolic blood pressure (Table 3).

**Table 2:** Mendelian randomization estimates of associations of genetically predicted linoleic acid with lipids, blood pressure, and reticulocyte count using different SNP selections

Outcome	Selection of SNPs	Method	#SNPs	Beta	95% CI	*p*
LDL cholesterol	Genome-wide significant and functionally relevant SNPs	PCA	47	*− 0.032*	*− 0.037 to − 0.027*	*< 0.001*
	Most significant SNPs	IVW	3	*− 0.034*	*− 0.039 to − 0.029*	*< 0.001*
	All genome-wide significant SNPs	PCA	167	*− 0.033*	*− 0.038 to − 0.028*	*< 0.001*
HDL cholesterol	Genome-wide significant and functionally relevant SNPs	PCA	47	*− 0.026*	*− 0.031 to − 0.021*	*< 0.001*
	Most significant SNPs	IVW^†^	3	*− 0.028*	*− 0.039 to − 0.016*	*< 0.001*
	All genome-wide significant SNPs*	PCA	141	*− 0.027*	*−0.032 to − 0.022*	*< 0.001*
Total cholesterol	Genome-wide significant and functionally relevant SNPs	PCA	47	*− 0.030*	*−0.035 to − 0.026*	*< 0.001*
	Most significant SNPs	IVW^†^	3	*− 0.032*	*− 0.040 to − 0.023*	*< 0.001*
	All genome-wide significant SNPs	PCA	167	*− 0.028*	*− 0.047 to − 0.010*	*0.003*
DBP	Genome-wide significant and functionally relevant SNPs	PCA	47	*0.006*	*0.003 to 0.009*	*< 0.001*
	Most significant SNPs	IVW^†^	3	0.008	− 0.005 to 0.020	0.23
	All genome-wide significant SNPs*	PCA	141	*0.006*	*0.003 to 0.009*	*< 0.001*
SBP	Genome-wide significant and functionally relevant SNPs	PCA	47	− 0.001	− 0.004 to 0.002	0.62
	Most significant SNPs	IVW^†^	3	0.001	− 0.008 to 0.009	0.90
	All genome-wide significant SNPs	PCA	167	0.010	− 0.007 to 0.027	0.24
Reticulocyte count	Genome-wide significant and functionally relevant SNPs	PCA	47	0.001	− 0.022 to 0.024	0.93
	Most significant SNPs	IVW^†^	3	0.013	− 0.001 to 0.027	0.08
	All genome-wide significant SNPs*	PCA	141	0.002	− 0.020 to 0.023	0.88

*DBP* diastolic blood pressure, *HDL* high-density lipoprotein, *IVW* inverse variance weighting, *LDL* low-density lipoprotein, *PCA* principal components analysis, *SBP* systolic blood pressure

*Twenty-six highly correlated SNPs were excluded due to potential pleiotropy with HDL cholesterol, DBP, and reticulocyte count

^†^IVW with random effects (heterogeneity test *p* value < 0.05) was used

**Table 3:** Sensitivity analysis of genetically predicted linoleic acid with ischemic heart disease and its risk factors using different analytic methods

Outcome	Methods	Odds ratio	95% CI	*p*	MR Egger intercept *p*
IHD	WM	0.98	0.96 to 1.01	0.09	
	MR Egger	0.97	0.94 to 1.01	0.10	0.32
Diabetes	WM	*0.97*	*0.95 to 0.99*	*0.001*	
	MR Egger	*0.95*	*0.93 to 0.98*	*0.001*	0.12
		Beta	95% CI		
LDL cholesterol	WM	*− 0.034*	*− 0.039 to − 0.029*	*< 0.001*	
	MR Egger	*− 0.035*	*− 0.055 to − 0.014*	*0.001*	0.95
HDL cholesterol	WM	*− 0.027*	*− 0.037 to − 0.016*	*0.01*	
	MR Egger	*− 0.017*	*− 0.033 to − 0.002*	*0.03*	0.12
Total cholesterol	WM	*− 0.032*	*− 0.043 to − 0.020*	*0.01*	
	MR Egger	*−0.032*	*−0.054 to − 0.010*	*0.004*	0.99
DBP	WM	0.006	− 0.001 to 0.013	0.07	
	MR Egger	− 0.002	− 0.018 to 0.015	0.85	0.15
SBP	WM	0.000	− 0.007 to 0.007	0.94	
	MR Egger	− 0.006	− 0.012 to 0.000	0.05	0.01
Reticulocyte count	WM	*0.012*	*0.005 to 0.019*	*0.02*	
	MR Egger	0.009	− 0.022 to 0.040	0.56	0.76

*DBP* diastolic blood pressure, *HDL* high-density lipoprotein, *LDL*, low-density lipoprotein, *SBP* systolic blood pressure, *WM* weighted median

LA was associated with higher diastolic blood pressure when using all genome-wide significant SNPs and functionally relevant SNPs, but this was not replicated using SNPs with top significance (Table 2). LA was not associated with systolic blood pressure (Table 2). LA was associated with higher reticulocyte count in sensitivity analysis using a weighted median (Table 3).

## Discussion

Using MR to obtain unconfounded estimates, our novel study shows an inverse association of LA with diabetes and lipids. Our study, together with a previous cohort study [8], suggests a benefit of LA for diabetes; the inverse association with lipids is also consistent with the cholesterol-lowering effect of LA. However, the benefit for IHD remains to be confirmed, consistent with the mixed findings from RCTs and meta-analysis of RCTs [4, 11, 12]. The associations with blood pressure and reticulocyte count are less clear; however, a positive association cannot be excluded.

To our knowledge, our study is the first MR study examining the effect of LA on IHD, diabetes, lipids, blood pressure, and reticulocyte count. Using genetic variants as proxies for LA, MR is less likely to be affected by the residual confounding and reverse causality inherent in observational studies. Moreover, the metabolism of n-6 PUFA interacts with that of n-3 PUFA [2], and our study applying MR to large publicly available data enables us to examine the independent effect of LA on IHD and its risk factors, in a cost-efficient way [39]. The IHD case-control study with over 70,000 cases and 260,000 controls, at an approximated *R*^2^ of 0.15 (percentage of variance explained by the three SNPs with top significance) in the GWAS of LA [16], has 0.8 power to detect an odds ratio (OR) of about 0.96 per percentage in total fatty acid increase in LA [40].

Nevertheless, several limitations exist. MR is based on three assumptions, i.e., the genetic instruments are associated with the exposure, no confounders of the associations of the genetic instruments with the outcomes exist, and the genetic variants are not linked with the outcomes other than via the relevant exposure (no pleiotropy) [13]. To satisfy these assumptions, we only selected SNPs strongly associated with LA, in functionally relevant genes, and also SNPs with top significance. We used PCA to use all the SNPs, a method that does not suffer from numerical instabilities arising from the potentially arbitrary SNP selection [20]. In addition, the sample for genetic variants on LA and for IHD and its risk factors only slightly (~ 1%) overlaps. As such, any correlation of the genetic variants with unmeasured confounders in the sample with LA is unlikely to be replicated in the samples with IHD and its risk factors, due to the different data structures [41]. We checked for known pleiotropy and used MR Egger to detect unknown pleiotropy. Given that population stratification might affect MR estimates, we obtained all the genetic associations from studies in people of European ancestry and with genomic control. As such, the associations might not apply to other populations. However, causal effects are not expected to vary by setting [42], although they would not be detectable or relevant in populations that do not consume vegetable oils. In addition, we could not assess whether associations varied by baseline level of LA. However, meta-analyses of the associations of LA with IHD do not suggest heterogeneity due to geographic locations with different baseline levels of LA [12]. A potential nonlinear association of serum LA with IHD could not be evaluated using summary data; to assess non-linearity needs individual-level data [43]. The influence of genetic predictors might be damped or buffered by compensatory developmental processes or feedback mechanisms [13]. However, such feedback mechanisms would be expected to mitigate the genetic effects, thus biasing toward the null, which would not explain the inverse associations of LA with diabetes and lipids in our study.

The effect of endogenous LA could differ from the effect of dietary LA. However, our findings have some consistency with RCTs specifically using LA as a dietary intervention, such as the Sydney Diet Heart Study and the Minnesota Coronary Experiment [10, 11], and their meta-analysis [11], as well as with the well-established cholesterol-lowering effect of dietary LA [10]. Using genetic instruments of objective biomarkers, i.e., serum LA, also minimizes measurement error arising from self-reported dietary consumption in nutrition studies. Our study could be affected by survivor bias (selection bias); however, the samples for LA and outcomes were not in the age range (75+ years) where survivor bias is thought to have a substantial effect [44]. The small effect size may not be clinically significant and represents the effect of lifetime exposure. MR estimates are less precise although less confounded than those from conventional observational studies [45]. However, relatively small effects of causal factors may still be an important determinant of population health, particularly for dietary factors which are modifiable and commonly consumed. We cannot exclude the possibility of reverse causality; however, it is unlikely that SNPs in genes related to the metabolism of LA affect LA via cardiovascular disease risk factors. A bi-directional MR is not feasible due to the lack of availability of genetic variants associated with LA. In addition, we could not assess whether the effect of LA on IHD and its risk factors varies by sex or age using the data freely available. As such, our estimates are likely to be conservative as some associations could be sex-specific, but the directions should not be reversed.

Notably, our novel study suggests a benefit of LA in diabetes and lipids, but that the benefit may not translate into a clear benefit for IHD. These paradoxical findings might also just be chance, which will be clarified by the use of stronger instruments and larger studies of IHD. However, a similar pattern has also occurred for other interventions targeting lipids, such as the cholesteryl ester transfer proteins, which lowered cholesterol and diabetes, but had collectively an unexpectedly null effect on IHD and an off-target hypertensive effect [46], possibly mediated by steroidogenesis [46, 47]. As such, a more general explanation with a mechanism underlying such a paradox might exist, given androgens are increasingly realized to affect cardiovascular risk [48, 49] as well as to improve glucose metabolism. Clarifying the underlying pathway would be beneficial for identifying effective new interventions for both diabetes and IHD.

## Conclusions

Our novel study suggests a benefit of LA for diabetes, LDL cholesterol, and total cholesterol, but no benefit for blood pressure or reticulocyte count. The benefit for IHD remains to be confirmed. Clarifying the role of LA and its underlying pathways would be worthwhile, with relevance to dietary recommendations and the identification of effective new interventions for both diabetes and IHD.

## Additional file

Additional file 1: **Table S1.** Characteristics of all genome-wide significant genetic predictors for linoleic acid (LA). (DOCX 64 kb)

## Abbreviations

CVD: Cardiovascular disease
DHA: Docosahexaenoic acid
DPA: Docosapentaenoic acid
EPA: Eicosapentaenoic acid
GWAS: Genom-wide association study
HDL: High-density lipoprotein
IHD: Ischemic heart disease
IVW: Inverse variance weighting
LA: Linoleic acid
LDL: Low-density lipoprotein
MR: Mendelian randomization
OR: Odds ratio
PCA: Principal components analysis
PUFA: Polyunsaturated fatty acid
RCTs: Randomized controlled trials
SNP: Single nucleotide polymorphism
