# Supplementary material for: Effect of linoleic acid on ischemic heart disease and its risk factors: a Mendelian randomization study
Source: BMC Med. 2019 Mar 14;17:61. doi: 10.1186/s12916-019-1293-x (PMC6417131; doi:10.1186/s12916-019-1293-x)
Supplement: Supplementary file 1 — Table S1. Characteristics of all genome-wide significant genetic predictors for linoleic acid (LA). (DOCX 64 kb) [file 12916_2019_1293_MOESM1_ESM.docx]

**Table S1 Characteristics of all genome-wide significant genetic predictors for linoleic acid (LA)**

| SNP | Gene | Effect allele | Association with LA | | F-statistic | Association with key confounders in the UK Biobank | | | | | |
| --- | --- | --- | --- | --- | --- | --- | --- | --- | --- | --- | --- |
|  |  |  | beta | *p* |  | Townsend index | Job type | Smoking status | Alcohol intake frequency | Moderate physical activity frequency | Vigorous physical activity frequency |
| rs174550 | *FADS1* | T | -1.47 | 4.37E-274 | 1447.6 | 0.0003 | 0.15 | 0.21 | 0.62 | 0.24 | 0.006 |
| rs174547 | *FADS1* | T | -1.47 | 4.98E-274 | 1447.3 | 0.0002 | 0.16 | 0.24 | 0.74 | 0.23 | 0.006 |
| rs174546 | *FADS1* | T | 1.47 | 2.13E-273 | 1443.8 | 0.0003 | 0.14 | 0.22 | 0.64 | 0.25 | 0.006 |
| rs174545 | *FADS1* | C | -1.47 | 4.37E-273 | 1442.2 | 0.0003 | 0.15 | 0.22 | 0.67 | 0.23 | 0.005 |
| rs174541 | *FADS1* | T | -1.45 | 4.90E-258 | 1360.7 | 0.0003 | 0.15 | 0.18 | 0.81 | 0.27 | 0.005 |
| rs174548 | *FADS1* | C | -1.42 | 2.14E-232 | 1222.1 | 0.0004 | 0.07 | 0.24 | 0.55 | 0.14 | 0.002 |
| rs174549 | *FADS1* | A | 1.43 | 3.88E-232 | 1220.7 | 0.001 | 0.12 | 0.28 | 0.54 | 0.22 | 0.004 |
| rs174555 | *FADS1* | T | -1.42 | 1.57E-231 | 1217.4 | 0.001 | 0.12 | 0.29 | 0.59 | 0.24 | 0.004 |
| rs174556 | *FADS1* | T | 1.39 | 2.43E-227 | 1194.8 | 0.001 | 0.12 | 0.30 | 0.56 | 0.22 | 0.003 |
| rs1535 | *FADS2* | A | -1.46 | 4.47E-272 | 1436.7 | 0.0002 | 0.15 | 0.20 | 0.92 | 0.25 | 0.006 |
| rs174574 | *FADS2* | A | 1.46 | 1.90E-267 | 1411.6 | 0.0001 | 0.12 | 0.17 | 0.99 | 0.19 | 0.003 |
| rs174576 | *FADS2* | A | 1.47 | 6.83E-266 | 1403.2 | 0.0003 | 0.12 | 0.22 | 0.91 | 0.21 | 0.004 |
| rs174578 | *FADS2* | A | 1.47 | 2.61E-263 | 1389.2 | 0.0002 | 0.12 | 0.21 | 0.83 | 0.20 | 0.004 |
| rs174583 | *FADS2* | T | 1.47 | 2.63E-263 | 1389.2 | 0.0003 | 0.10 | 0.38 | 0.90 | 0.21 | 0.003 |
| rs174577 | *FADS2* | A | 1.46 | 1.73E-262 | 1384.8 | 0.0002 | 0.11 | 0.22 | 0.86 | 0.20 | 0.004 |
| rs174601 | *FADS2* | T | 1.57 | 2.82E-253 | 1335.0 | 0.001 | 0.10 | 0.46 | 0.78 | 0.22 | 0.002 |
| rs174570 | *FADS2* | T | 1.56 | 9.98E-149 | 771.4 | 0.12 | 0.37 | 0.16 | 0.50 | 0.36 | 0.05 |
| rs2727270 | *FADS2* | T | 1.62 | 1.79E-142 | 737.8 | 0.18 | 0.02 | 0.32 | 0.40 | 0.08 | 0.01 |
| rs2727271 | *FADS2* | A | -1.61 | 3.93E-142 | 736.0 | 0.18 | 0.02 | 0.32 | 0.40 | 0.09 | 0.01 |
| rs2072114 | *FADS2* | A | -1.56 | 6.13E-141 | 729.6 | 0.24 | 0.04 | 0.27 | 0.72 | 0.06 | 0.01 |
| rs2524299 | *FADS2* | A | -1.58 | 1.80E-138 | 716.3 | 0.12 | 0.01 | 0.26 | 0.35 | 0.04 | 0.004 |
| rs2845573 | *FADS2* | A | -1.74 | 1.92E-116 | 598.4 | 0.22 | 0.17 | 0.23 | 0.18 | 0.61 | 0.08 |
| rs174575 | *FADS2* | C | -1.09 | 4.07E-114 | 585.9 | 0.001 | 0.38 | 0.47 | 0.41 | 0.32 | 0.04 |
| rs2526678 | *FADS2* | A | 1.77 | 9.18E-106 | 541.3 | 0.52 | 0.16 | 0.61 | 0.17 | 0.41 | 0.08 |
| rs2851682 | *FADS2* | A | -1.61 | 3.67E-103 | 527.4 | 0.49 | 0.10 | 0.64 | 0.12 | 0.35 | 0.05 |
| rs174611 | *FADS2* | T | -0.93 | 1.51E-89 | 454.9 | 0.02 | 0.36 | 0.51 | 0.50 | 0.16 | 0.02 |
| rs174605 | *FADS2* | T | 0.92 | 2.07E-86 | 438.2 | 0.01 | 0.32 | 0.55 | 0.49 | 0.30 | 0.02 |
| rs174591 | *FADS2* | A | 1 | 2.31E-86 | 438.0 | 0.001 | 0.53 | 0.71 | 0.20 | 0.47 | 0.10 |
| rs174616 | *FADS2* | A | 0.79 | 1.24E-79 | 402.2 | 0.01 | 0.92 | 0.67 | 0.27 | 0.60 | 0.06 |
| rs174579 | *FADS2* | T | 0.93 | 4.34E-72 | 362.2 | 0.002 | 0.89 | 0.68 | 0.82 | 0.75 | 0.24 |
| rs174626 | *FADS2* | A | -0.74 | 7.59E-71 | 355.6 | 0.01 | 0.95 | 0.97 | 0.48 | 0.59 | 0.10 |
| rs174593 | *FADS2* | T | -1.05 | 1.58E-69 | 348.7 | 0.001 | 0.48 | 0.71 | 0.08 | 0.54 | 0.06 |
| rs174589 | *FADS2* | C | -0.94 | 1.76E-69 | 348.4 | 0.001 | 0.93 | 0.67 | 0.71 | 0.56 | 0.40 |
| rs174597 | *FADS2* | C | 1.06 | 1.77E-69 | 348.4 | 0.001 | 0.81 | 0.34 | 0.33 | 0.74 | 0.08 |
| rs526126 | *FADS2* | C | -1.05 | 5.34E-55 | 272.0 | 0.03 | 0.31 | 0.86 | 0.38 | 0.40 | 0.31 |
| rs968567 | *FADS2* | T | 0.76 | 7.59E-43 | 208.3 | 0.01 | 0.82 | 0.70 | 0.78 | 0.89 | 0.22 |
| rs174627 | *FADS2* | A | 0.7 | 2.04E-33 | 159.2 | 0.05 | 0.55 | 0.34 | 0.45 | 0.69 | 0.39 |
| rs17764324 | *FADS2* | T | 0.9 | 3.83E-32 | 152.6 | 0.53 | 0.96 | 0.42 | 0.01 | 0.04 | 0.05 |
| rs17831757 | *FADS2* | T | -0.9 | 5.33E-32 | 151.9 | 0.54 | 0.97 | 0.41 | 0.01 | 0.05 | 0.05 |
| rs11230815 | *FADS2* | C | -0.9 | 7.03E-32 | 151.2 | 0.55 | 0.98 | 0.37 | 0.01 | 0.04 | 0.04 |
| rs7935946 | *FADS2* | T | 1.18 | 8.16E-24 | 109.7 | 0.94 | 0.13 | 0.72 | 0.12 | 0.03 | 0.12 |
| rs17156442 | *FADS2* | T | 1.18 | 1.94E-21 | 97.6 | 0.88 | 0.12 | 0.72 | 0.12 | 0.03 | 0.14 |
| rs482548 | *FADS2* | T | -0.46 | 3.26E-11 | 46.3 | 0.98 | 0.88 | 0.31 | 0.02 | 0.0003 | 0.26 |
| rs16966952 | *NTAN1* | A | 0.35 | 1.23E-15 | 68.3 | 0.004 | 0.39 | 0.58 | 0.99 | 0.02 | 0.03 |
| rs3803573 | *NTAN1* | T | 0.35 | 1.39E-15 | 68.0 | 0.005 | 0.40 | 0.66 | 0.96 | 0.02 | 0.03 |
| rs4985148 | *NTAN1* | A | -0.34 | 1.21E-14 | 63.3 | 0.01 | 0.39 | 0.62 | 0.87 | 0.04 | 0.10 |
| rs4500751 | *NTAN1* | T | 0.34 | 1.47E-14 | 62.9 | 0.02 | 0.46 | 0.74 | 0.97 | 0.05 | 0.09 |
| rs2727266 | *BEST1* | A | -0.72 | 7.31E-17 | 74.4 | 0.70 | 0.39 | 0.97 | 0.47 | 0.07 | 0.25 |
| rs1800009 | *BEST1* | T | -0.34 | 1.63E-14 | 62.7 | 0.005 | 0.53 | 0.64 | 0.11 | 0.98 | 0.27 |
| rs2727261 | *BEST1* | T | 0.74 | 2.21E-13 | 57.0 | 0.16 | 0.13 | 0.78 | 0.17 | 0.70 | 0.94 |
| rs1109748 | *BEST1* | A | 0.72 | 6.47E-12 | 49.8 | 0.41 | 0.46 | 0.10 | 0.23 | 0.82 | 0.82 |
| rs2521572 | *BEST1* | T | 0.86 | 7.49E-12 | 49.5 | 0.78 | 0.67 | 0.43 | 0.27 | 0.87 | 1.00 |
| rs760306 | *BEST1* | T | 0.28 | 1.46E-08 | 33.4 | 0.92 | 0.86 | 0.41 | 0.33 | 0.54 | 0.28 |
| rs17156609 | *BEST1* | A | 0.82 | 4.09E-08 | 31.3 | 0.003 | 0.93 | 0.26 | 0.43 | 0.52 | 0.83 |
| rs102275 | *C11orf10* | T | -1.46 | 3.01E-271 | 1432.2 | 0.0003 | 0.14 | 0.18 | 0.67 | 0.18 | 0.003 |
| rs174538 | *C11orf10* | A | 1.43 | 4.21E-228 | 1198.9 | 0.001 | 0.45 | 0.19 | 0.94 | 0.55 | 0.01 |
| rs174535 | *C11orf9* | T | -1.48 | 1.04E-273 | 1445.5 | 0.001 | 0.13 | 0.32 | 0.60 | 0.26 | 0.01 |
| rs174536 | *C11orf9* | A | -1.48 | 1.09E-273 | 1445.4 | 0.001 | 0.16 | 0.34 | 0.54 | 0.27 | 0.01 |
| rs174537 | *C11orf9* | T | 1.47 | 1.18E-271 | 1434.4 | 0.001 | 0.17 | 0.23 | 0.58 | 0.32 | 0.01 |
| rs174528 | *C11orf9* | T | -1.43 | 3.05E-249 | 1313.2 | 0.003 | 0.12 | 0.79 | 0.43 | 0.34 | 0.01 |
| rs174534 | *C11orf9* | A | -1.37 | 1.89E-208 | 1092.8 | 0.01 | 0.48 | 0.76 | 0.76 | 0.97 | 0.03 |
| rs108499 | *C11orf9* | T | 1.38 | 2.36E-208 | 1092.3 | 0.01 | 0.46 | 0.95 | 0.72 | 0.91 | 0.03 |
| rs174532 | *C11orf9* | A | -1.05 | 1.08E-89 | 455.7 | 0.01 | 0.32 | 0.36 | 0.03 | 0.03 | 0.44 |
| rs509360 | *C11orf9* | A | -0.93 | 9.52E-82 | 413.4 | 0.43 | 0.70 | 0.57 | 0.27 | 0.005 | 0.07 |
| rs149803 | *C11orf9* | C | 1.13 | 1.46E-80 | 407.1 | 0.06 | 0.31 | 0.26 | 0.01 | 0.02 | 0.56 |
| rs2269928 | *C11orf9* | T | -1.15 | 3.65E-62 | 309.7 | 0.07 | 0.39 | 0.94 | 0.89 | 0.59 | 0.89 |
| rs650436 | *C11orf9* | T | -0.52 | 9.68E-29 | 135.0 | 0.09 | 0.87 | 0.04 | 0.08 | 0.30 | 0.91 |
| rs579383 | *C11orf9* | A | 0.49 | 2.81E-26 | 122.4 | 0.08 | 0.86 | 0.08 | 0.10 | 0.28 | 0.95 |
| rs198476 | *C11orf9* | A | -0.44 | 2.94E-26 | 122.3 | 0.16 | 0.81 | 0.15 | 0.19 | 0.16 | 0.59 |
| rs198462 | *C11orf9* | A | -0.44 | 3.97E-26 | 121.6 | 0.18 | 0.80 | 0.14 | 0.26 | 0.21 | 0.61 |
| rs198464 | *C11orf9* | A | -0.44 | 5.05E-26 | 121.1 | 0.14 | 0.66 | 0.18 | 0.25 | 0.17 | 0.59 |
| rs569258 | *C11orf9* | T | 0.4 | 9.01E-21 | 94.2 | 0.12 | 0.28 | 0.00 | 0.01 | 0.24 | 0.48 |
| rs198473 | *C11orf9* | A | -0.39 | 8.31E-14 | 59.1 | 0.51 | 0.39 | 0.53 | 0.58 | 0.76 | 0.75 |
| rs198475 | *C11orf9* | T | 0.38 | 1.14E-13 | 58.5 | 0.48 | 0.38 | 0.52 | 0.57 | 0.71 | 0.73 |
| rs198426 | *DAGLA* | T | -0.39 | 2.43E-19 | 87.0 | 0.07 | 0.59 | 0.001 | 0.03 | 0.07 | 0.25 |
| rs1692120 | *DAGLA* | A | -0.33 | 1.12E-14 | 63.5 | 0.004 | 0.20 | 0.71 | 0.67 | 0.01 | 0.95 |
| rs2453710 | *DAGLA* | A | 0.31 | 1.35E-12 | 53.1 | 0.05 | 0.05 | 0.53 | 0.61 | 0.03 | 0.77 |
| rs198428 | *DAGLA* | A | -0.31 | 1.79E-12 | 52.5 | 0.16 | 0.36 | 0.01 | 0.02 | 0.18 | 0.18 |
| rs11230767 | *DAGLA* | A | -0.28 | 7.59E-11 | 44.5 | 0.002 | 0.69 | 0.11 | 0.62 | 0.0002 | 0.24 |
| rs11230766 | *DAGLA* | A | 0.28 | 9.91E-11 | 43.9 | 0.003 | 0.67 | 0.11 | 0.63 | 0.0002 | 0.24 |
| rs198436 | *DAGLA* | T | 0.28 | 3.71E-10 | 41.1 | 0.18 | 0.39 | 0.02 | 0.02 | 0.34 | 0.22 |
| rs12794220 | *DAGLA* | T | -0.27 | 5.14E-10 | 40.5 | 0.22 | 0.28 | 0.003 | 0.01 | 0.27 | 0.21 |
| rs198456 | *DAGLA* | A | -0.26 | 1.41E-09 | 38.3 | 0.15 | 0.36 | 0.03 | 0.02 | 0.34 | 0.23 |
| rs12281961 | *DAGLA* | T | 0.27 | 8.50E-09 | 34.5 | 0.04 | 0.35 | 0.05 | 0.41 | 0.002 | 0.14 |
| rs4335555 | *DAGLA* | C | 0.26 | 9.42E-09 | 34.3 | 0.03 | 0.58 | 0.04 | 0.27 | 0.003 | 0.12 |
| rs1812458 | *DAGLA* | T | 0.26 | 1.12E-08 | 34.0 | 0.04 | 0.49 | 0.04 | 0.27 | 0.002 | 0.11 |
| rs962371 | *DAGLA* | T | 0.26 | 1.17E-08 | 33.9 | 0.04 | 0.51 | 0.04 | 0.26 | 0.002 | 0.11 |
| rs12284414 | *DAGLA* | A | -0.27 | 1.23E-08 | 33.8 | 0.04 | 0.54 | 0.04 | 0.26 | 0.002 | 0.12 |
| rs17626916 | *DAGLA* | A | -0.26 | 1.44E-08 | 33.4 | 0.09 | 0.34 | 0.04 | 0.41 | 0.002 | 0.15 |
| rs12806760 | *DAGLA* | A | -0.27 | 1.54E-08 | 33.3 | 0.09 | 0.31 | 0.04 | 0.41 | 0.002 | 0.14 |
| rs1692126 | *DAGLA* | C | -0.26 | 2.87E-08 | 32.0 | 0.07 | 0.13 | 0.06 | 0.76 | 0.001 | 0.12 |
| rs174448 | *FADS3* | A | -0.97 | 1.15E-111 | 572.8 | 0.005 | 0.57 | 0.47 | 0.70 | 0.11 | 0.02 |
| rs174449 | *FADS3* | A | -0.96 | 3.37E-110 | 565.0 | 0.01 | 0.75 | 0.65 | 0.58 | 0.22 | 0.05 |
| rs422249 | *FADS3* | T | 0.98 | 1.94E-103 | 528.9 | 0.02 | 0.53 | 0.49 | 0.76 | 0.08 | 0.01 |
| rs174455 | *FADS3* | A | -0.93 | 1.17E-98 | 503.4 | 0.004 | 1.00 | 0.77 | 0.55 | 0.06 | 0.01 |
| rs174450 | *FADS3* | T | -0.76 | 1.12E-74 | 375.9 | 0.01 | 0.96 | 0.90 | 0.65 | 0.73 | 0.14 |
| rs174634 | *FADS3* | C | -0.74 | 3.83E-52 | 257.0 | 0.001 | 1.00 | 0.47 | 0.61 | 0.73 | 0.25 |
| rs174464 | *FADS3* | A | 0.74 | 2.41E-51 | 252.8 | 0.001 | 0.86 | 0.42 | 0.50 | 0.75 | 0.19 |
| rs174456 | *FADS3* | A | -0.73 | 2.41E-51 | 252.8 | 0.001 | 0.88 | 0.42 | 0.48 | 0.76 | 0.22 |
| rs1000778 | *FADS3* | A | 0.72 | 3.10E-50 | 247.0 | 0.001 | 0.88 | 0.41 | 0.46 | 0.76 | 0.22 |
| rs7104849 | *FADS3* | A | -0.89 | 1.28E-31 | 149.9 | 0.48 | 0.87 | 0.40 | 0.01 | 0.02 | 0.02 |
| rs7394871 | *FADS3* | A | 1.58 | 2.94E-31 | 148.0 | 0.81 | 0.86 | 0.29 | 0.05 | 0.002 | 0.02 |
| rs7482316 | *FADS3* | A | -0.86 | 6.41E-30 | 141.1 | 0.95 | 0.78 | 0.35 | 0.01 | 0.02 | 0.05 |
| rs472031 | *FADS3* | A | -0.48 | 9.57E-12 | 48.9 | 0.88 | 0.95 | 0.43 | 0.05 | 0.0005 | 0.23 |
| rs4246215 | *FEN1* | T | 1.45 | 1.19E-257 | 1358.6 | 0.0003 | 0.15 | 0.16 | 0.74 | 0.34 | 0.01 |
| rs412334 | *FEN1* | T | -0.98 | 6.24E-47 | 229.6 | 0.05 | 0.76 | 0.91 | 0.09 | 0.64 | 0.09 |
| rs695867 | *FEN1* | A | -1.05 | 5.73E-20 | 90.1 | 0.43 | 0.87 | 0.47 | 0.84 | 0.98 | 0.99 |
| rs3758977 | *FTH1* | T | -0.34 | 3.81E-14 | 60.8 | 0.005 | 0.44 | 0.60 | 0.14 | 0.99 | 0.22 |
| rs2028062 | *FTH1* | A | 0.33 | 9.27E-14 | 58.9 | 0.01 | 0.59 | 0.51 | 0.25 | 0.92 | 0.20 |
| rs10792320 | *FTH1* | A | -0.33 | 1.44E-13 | 58.0 | 0.01 | 0.73 | 0.55 | 0.33 | 0.93 | 0.20 |
| rs11230874 | *FTH1* | T | -0.82 | 1.07E-09 | 38.9 | 0.80 | 0.96 | 0.15 | 0.28 | 0.24 | 0.73 |
| rs10897208 | *FTH1* | A | -0.79 | 4.58E-09 | 35.8 | 0.81 | 0.88 | 0.15 | 0.30 | 0.24 | 0.76 |
| rs17156618 | *FTH1* | A | -0.82 | 4.61E-08 | 31.0 | 0.003 | 0.77 | 0.31 | 0.67 | 0.44 | 0.88 |
| rs4963466 | *INCENP* | C | -0.3 | 6.02E-10 | 40.1 | 0.03 | 0.79 | 0.07 | 0.63 | 0.01 | 0.71 |
| rs3781974 | *INCENP* | A | 0.29 | 6.65E-10 | 39.9 | 0.04 | 0.73 | 0.05 | 0.67 | 0.01 | 0.66 |
| rs1460027 | *INCENP* | A | 0.28 | 4.50E-09 | 35.9 | 0.04 | 0.67 | 0.05 | 0.66 | 0.03 | 0.31 |
| rs11230941 | *INCENP* | A | 0.27 | 8.17E-09 | 34.6 | 0.04 | 0.58 | 0.07 | 0.70 | 0.04 | 0.36 |
| rs2903922 | *INCENP* | A | 0.27 | 9.25E-09 | 34.4 | 0.05 | 0.56 | 0.06 | 0.62 | 0.03 | 0.39 |
| rs11230896 | *INCENP* | T | -0.33 | 1.04E-08 | 34.1 | 0.09 | 0.94 | 0.72 | 0.98 | 0.95 | 0.29 |
| rs7080386 | *JMJD1C* | A | -0.25 | 6.80E-09 | 35.0 | 0.22 | 0.02 | 0.003 | 0.15 | 0.16 | 0.60 |
| rs10740118 | *JMJD1C* | C | -0.25 | 8.08E-09 | 34.6 | 0.30 | 0.03 | 0.002 | 0.18 | 0.15 | 0.58 |
| rs10761741 | *JMJD1C* | T | -0.25 | 8.77E-09 | 34.5 | 0.28 | 0.03 | 0.002 | 0.19 | 0.15 | 0.56 |
| rs7075195 | *JMJD1C* | A | 0.25 | 9.02E-09 | 34.4 | 0.27 | 0.03 | 0.002 | 0.18 | 0.14 | 0.55 |
| rs10761731 | *JMJD1C* | A | 0.25 | 9.10E-09 | 34.4 | 0.30 | 0.03 | 0.003 | 0.19 | 0.17 | 0.59 |
| rs10761739 | *JMJD1C* | C | -0.25 | 9.78E-09 | 34.2 | 0.28 | 0.03 | 0.002 | 0.18 | 0.16 | 0.56 |
| rs7896518 | *JMJD1C* | A | 0.25 | 1.07E-08 | 34.1 | 0.27 | 0.04 | 0.002 | 0.15 | 0.16 | 0.67 |
| rs10761771 | *JMJD1C* | T | 0.23 | 3.69E-08 | 31.5 | 0.61 | 0.02 | 0.004 | 0.67 | 0.23 | 0.84 |
| rs10822163 | *JMJD1C* | C | 0.24 | 3.91E-08 | 31.4 | 0.62 | 0.02 | 0.003 | 0.69 | 0.24 | 0.78 |
| rs2393977 | *JMJD1C* | A | 0.24 | 4.04E-08 | 31.3 | 0.60 | 0.02 | 0.004 | 0.67 | 0.23 | 0.83 |
| rs7909960 | *JMJD1C* | A | -0.24 | 4.07E-08 | 31.3 | 0.60 | 0.02 | 0.004 | 0.68 | 0.22 | 0.84 |
| rs4454603 | *JMJD1C* | T | -0.23 | 4.25E-08 | 31.2 | 0.55 | 0.02 | 0.003 | 0.68 | 0.26 | 0.77 |
| rs10740129 | *JMJD1C* | A | -0.24 | 4.65E-08 | 31.0 | 0.60 | 0.02 | 0.004 | 0.69 | 0.23 | 0.82 |
| rs12355784 | *JMJD1C* | A | -0.23 | 4.84E-08 | 30.9 | 0.60 | 0.02 | 0.003 | 0.69 | 0.23 | 0.82 |
| rs10761742 | *JMJD1C* | A | 0.24 | 4.98E-08 | 30.8 | 0.55 | 0.02 | 0.004 | 0.65 | 0.24 | 0.75 |
| rs1136001 | *PDXDC1* | T | 0.35 | 9.86E-16 | 68.8 | 0.003 | 0.44 | 0.58 | 0.97 | 0.02 | 0.04 |
| rs6498540 | *PDXDC1* | A | -0.35 | 9.97E-16 | 68.7 | 0.004 | 0.45 | 0.62 | 0.80 | 0.02 | 0.04 |
| rs1741 | *PDXDC1* | C | 0.35 | 1.00E-15 | 68.7 | 0.002 | 0.48 | 0.61 | 0.94 | 0.02 | 0.03 |
| rs1121 | *PDXDC1* | A | 0.35 | 1.02E-15 | 68.7 | 0.003 | 0.40 | 0.55 | 0.99 | 0.02 | 0.03 |
| rs1135999 | *PDXDC1* | A | -0.35 | 1.06E-15 | 68.6 | 0.004 | 0.39 | 0.59 | 0.93 | 0.02 | 0.03 |
| rs2740 | *PDXDC1* | A | -0.35 | 1.10E-15 | 68.5 | 0.004 | 0.40 | 0.59 | 0.93 | 0.02 | 0.03 |
| rs7200543 | *PDXDC1* | A | -0.35 | 1.42E-15 | 68.0 | 0.003 | 0.38 | 0.58 | 0.98 | 0.02 | 0.03 |
| rs4985155 | *PDXDC1* | A | -0.34 | 2.85E-15 | 66.5 | 0.01 | 0.83 | 0.80 | 0.53 | 0.03 | 0.12 |
| rs3198697 | *PDXDC1* | T | -0.35 | 3.27E-15 | 66.2 | 0.35 | 0.17 | 0.20 | 0.55 | 0.07 | 0.22 |
| rs4985124 | *PDXDC1* | T | -0.34 | 1.33E-14 | 63.1 | 0.003 | 0.46 | 0.51 | 0.89 | 0.02 | 0.03 |
| rs174468 | *RAB3IL1* | A | -0.79 | 9.81E-64 | 318.0 | 0.01 | 0.05 | 0.41 | 0.72 | 0.90 | 0.02 |
| rs174476 | *RAB3IL1* | T | -0.76 | 2.34E-60 | 300.2 | 0.005 | 0.09 | 0.51 | 0.92 | 0.78 | 0.03 |
| rs666870 | *RAB3IL1* | A | -0.76 | 2.71E-60 | 299.9 | 0.01 | 0.09 | 0.49 | 0.89 | 0.75 | 0.02 |
| rs174478 | *RAB3IL1* | T | 0.76 | 2.84E-60 | 299.8 | 0.005 | 0.11 | 0.56 | 0.87 | 0.71 | 0.02 |
| rs174479 | *RAB3IL1* | C | -0.89 | 5.39E-41 | 198.6 | 0.01 | 0.84 | 0.36 | 0.47 | 0.58 | 0.16 |
| rs17764935 | *RAB3IL1* | A | 1.49 | 1.23E-31 | 150.0 | 0.64 | 0.69 | 0.77 | 0.06 | 0.0005 | 0.07 |
| rs2521568 | *RAB3IL1* | C | 0.72 | 7.39E-17 | 74.4 | 0.68 | 0.30 | 0.79 | 0.44 | 0.06 | 0.37 |
| rs174472 | *RAB3IL1* | A | -0.39 | 2.11E-14 | 62.1 | 0.01 | 0.02 | 0.24 | 0.56 | 0.002 | 0.70 |
| rs2235093 | *RAB3IL1* | A | -0.35 | 3.60E-12 | 51.0 | 0.004 | 0.17 | 0.43 | 0.61 | 0.003 | 0.77 |
| rs13966 | *RAB3IL1* | T | -0.33 | 6.35E-12 | 49.8 | 0.004 | 0.17 | 0.45 | 0.60 | 0.003 | 0.81 |
| rs7897379 | *REEP3* | T | 0.26 | 9.67E-09 | 34.3 | 0.51 | 0.01 | 0.02 | 0.78 | 0.31 | 0.81 |
| rs2393986 | *REEP3* | A | 0.25 | 2.67E-08 | 32.1 | 0.45 | 0.02 | 0.02 | 0.82 | 0.21 | 0.90 |
| rs10740134 | *REEP3* | T | 0.25 | 2.87E-08 | 32.0 | 0.46 | 0.01 | 0.02 | 0.81 | 0.22 | 0.88 |
| rs10761784 | *REEP3* | A | 0.25 | 3.01E-08 | 31.9 | 0.47 | 0.01 | 0.02 | 0.82 | 0.23 | 0.87 |
| rs7085018 | *REEP3* | T | 0.24 | 3.16E-08 | 31.8 | 0.55 | 0.02 | 0.02 | 0.77 | 0.29 | 0.78 |
| rs7920036 | *REEP3* | T | 0.24 | 3.59E-08 | 31.5 | 0.56 | 0.02 | 0.02 | 0.79 | 0.31 | 0.79 |
| rs10761779 | *REEP3* | A | 0.23 | 4.67E-08 | 31.0 | 0.60 | 0.02 | 0.004 | 0.70 | 0.27 | 0.79 |
| rs4486511 | *REEP3* | T | -0.23 | 4.68E-08 | 31.0 | 0.58 | 0.02 | 0.00 | 0.67 | 0.24 | 0.84 |
| rs10509189 | *REEP3* | T | 0.23 | 4.69E-08 | 31.0 | 0.59 | 0.02 | 0.004 | 0.69 | 0.25 | 0.81 |
| rs9971352 | *REEP3* | A | 0.23 | 4.70E-08 | 31.0 | 0.59 | 0.02 | 0.004 | 0.68 | 0.25 | 0.80 |
| rs2298823 | *SCGB1D1* | C | -0.27 | 3.03E-08 | 31.9 | 0.04 | 0.82 | 0.05 | 0.74 | 0.06 | 0.24 |
| rs1675102 | *SCGB1D1* | T | 0.26 | 4.29E-08 | 31.2 | 0.13 | 0.59 | 0.09 | 1.00 | 0.05 | 0.19 |
| rs10897169 | *SYT7* | A | -0.27 | 1.52E-08 | 33.3 | 0.04 | 0.38 | 0.06 | 0.43 | 0.001 | 0.15 |

^*^The p values were obtained from the UK Biobank summary data that genotyped ~850,000 variants for over 4,000 phenotypes. None of the genetic variants reached Bonferroni-corrected significance. The SNPs in *FADS1*, *FADS2* and *NTAN1* are functionally relevant SNPs used in the study.
